# Ciliary Neurotrophic Factor in Skeletal Muscle of Older Adults and the Effects of Weight Loss and Aerobic Training

**DOI:** 10.3390/metabo16080596

**Published:** 2026-08-21

**Authors:** Alice S. Ryan, Guoyan Li, Colleen Lynch, Sausan M. Jaber, Galya Bigman

**Affiliations:** 1 VA Research Service, VA Maryland Health Care System, 10 N Greene Street, Baltimore, MA 21201, USA; 2 Department of Medicine, Division of Gerontology, Geriatrics, and Palliative Care, University of Maryland School of Medicine, 655 W Baltimore Street, Baltimore, MA 21201, USA; 3 Baltimore VA Medical Center Geriatric Research, Education and Clinical Center, 10 N Greene Street, Baltimore, MA 21201, USA; 4 Department of Epidemiology, University of Maryland School of Medicine, 655 W Baltimore Street, Baltimore, MA 21201, USA; gbigman@som.umaryland.edu

**Keywords:** ciliary neurotrophic factor, skeletal muscle, older adults, insulin sensitivity, glucose tolerance, weight loss, aerobic exercise, obesity

## Abstract

**Background/Objectives:** Ciliary neurotrophic factor (CNTF), a pleiotropic cytokine with central and peripheral actions, has been implicated in obesity, insulin resistance, and aging, yet its role in skeletal muscle and cardiometabolic health remains unclear. This study examined skeletal muscle CNTF mRNA expression in relation to adiposity and glucose tolerance in older adults and assessed the effects of six-month weight loss (WL) and aerobic exercise training (AEX) interventions on its expression. **Methods:** Sixty-seven adults (age, 60.75 ± 7.58) underwent a vastus lateralis muscle biopsy, VO_2_max testing, dual-energy X-ray absorptiometry (DXA) scan, a resting metabolic rate (RMR) and substrate oxidation assessment, and a 3-h oral glucose tolerance test with glucose and insulin area under the curve (AUC) calculation. A subset completed 6 months of either a WL program (*n* = 21) or a supervised 3 times per week AEX intervention (*n* = 20). **Results:** At baseline, skeletal muscle CNTF expression was inversely associated with RMR, fat oxidation, 3-h glucose AUC, and insulin AUC, but not with BMI, percent body fat, or VO_2_max. WL reduced body weight, fat mass, glucose AUC, insulin AUC, and basal muscle CNTF expression, whereas AEX increased VO_2_max and reduced fat mass without altering body weight, glucose AUC, insulin AUC, or basal CNTF. Insulin-stimulated CNTF expression increased after both WL and AEX, and the intervention-induced change in glucose disposal (M) was positively associated with the change in insulin-stimulated CNTF. **Conclusions:** In older adults, skeletal muscle CNTF is associated with glucose intolerance but is not associated with body composition or aerobic fitness. Improvements in insulin sensitivity are associated with increases in muscle CNTF during hyperinsulinemia. Further work is needed to determine the mechanism for muscle CNTF change during hyperinsulinemia after weight loss and exercise training, and its role in glucose metabolism and obesity in older adults.

## 1. Introduction

Ciliary neurotrophic factor (CNTF) is a pleiotropic cytokine that acts both centrally and peripherally to regulate energy metabolism and neuronal survival [1]. CNTF binds to the CNTFα receptor, driving heterodimerization of glycoprotein 130 (gp130) and the leukemia inhibitory factor receptor (LIFR), which activate downstream signaling through the JAK/STAT, PI3K/Akt, and Ras-MAPK pathways [2]. Through these pathways, CNTF reduces inflammatory signaling associated with lipid accumulation in the liver and skeletal muscle and increases glucose uptake in skeletal muscle in animal models [3]. CNTF has been proposed to act centrally to alter obesity, as administration of recombinant CNTF reduces leptin resistance, suppresses appetite, and induces weight loss in obese rodents [4]. Together, these central and peripheral actions position CNTF as a candidate therapeutic that may promote increased insulin sensitivity and fatty acid oxidation in peripheral tissues in the preclinical setting [1].

CNTF has also been implicated as having a myotrophic role and, when administered for one week to a mouse model of diet-induced obesity, resulted in muscle growth and energy expenditure [5]. In humans, CNTF measured in the plasma is higher in patients with obesity, with and without diabetes, than in healthy normal weight controls [6]. Moreover, circulating CNTF levels are associated with BMI, fasting glucose, fasting insulin, Homeostatic Model Assessment of Insulin Resistance (HOMA index), and inflammatory markers of C-reactive protein (CRP) and Interleukin-6 (IL-6) in older adults (~60 years of age) [6]. In a 12-week double-blind, randomized clinical trial, nondiabetic obese adults who received subcutaneous injections of recombinant human variant CNTF experienced more weight loss than controls [7], but almost 90% of participants produced neutralizing antibodies against the drug (Axokine) [7]. Although early trials may have shown promise for CNTF as an anti-obesity agent, alternative non-pharmacological strategies to alter CNTF levels could be considered.

Skeletal muscle has emerged as an important endocrine organ, secreting myokines that influence immunometabolism and glucose homeostasis. Compared with other, more extensively studied myokines, CNTF has received relatively less attention. Aging is associated with changes in muscle mass, mitochondrial function, and chronic inflammatory signaling—all of which may affect myokine expression. One mechanism by which CNTF is thought to exert its metabolic effects is by activating AMP-activated protein kinase (AMPK) in skeletal muscle [3,8]. AMPK functions as a cellular energy sensor, detecting the ratio of AMP/ADP to ATP [9]. If ATP levels drop and AMP levels rise, AMPK turns on and restores energy balance by upregulating ATP-generating pathways, including mitochondrial fatty acid oxidation and glucose uptake [9,10,11]. AMPK activation may therefore serve as a major mechanism by which CNTF improves peripheral insulin sensitivity and reverses insulin resistance in models of obesity and diabetes [11].

Aging may influence CNTF levels, as muscle mRNA expression of CNTF is higher in young (*n* = 15) than older (*n* = 16) healthy adults and does not change in the older adults after an acute bout of resistance exercise, suggesting an impaired response to exercise with aging [12]. In another study conducted by the same group of investigators and the only other exercise study that examined human muscle CNTF, the gain in leg extension strength after training was significantly related to baseline and post-training skeletal muscle CNTF gene expression in a small sample (*n* = 8) who underwent 36 sessions of resistance training [13]. Thus, little is known with regard to how skeletal muscle CNTF is affected by exercise or by weight loss in human trials.

The purpose of this study was to determine skeletal muscle CNTF mRNA expression during basal and insulin-stimulated in vivo conditions and its relationship to obesity, energy utilization, and insulin sensitivity in older adults, and in a subset, the effects of six-month weight loss (WL) and aerobic exercise training (AEX) interventions on its expression. Our central hypothesis was that skeletal muscle CNTF would be associated with glucose metabolism and energy expenditure and would decrease after interventions of weight loss or aerobic training.

## 2. Materials and Methods

Participants who enrolled were aged greater than 45 years, had a body mass index (BMI) > 25 kg/m^2^ but less than 50 kg/m^2^, were nonsmokers, were not on insulin, hypoglycemic agents, anti-inflammatory medications, or hormone replacement therapy, and were weight stable (<2 kg weight change in past year) and sedentary (<20 min of structured aerobic activity per week). Methods and procedures were approved by the Institutional Review Board at the University of Maryland School of Medicine and the VA Research and Development Committee. Each participant signed consent and was screened by a physical examination, medical history, and fasting blood profile.

### 2.1. Body Composition and Aerobic Fitness Testing

Participants had height (cm) and weight (kg) measured to calculate BMI. Body composition, including percent body fat, fat, and lean mass or fat-free mass (FFM), was determined by dual-energy X-ray absorptiometry (iDXA, LUNAR Radiation Corp., Madison, WI, USA). Aerobic fitness was assessed by maximal oxygen consumption (VO_2_max), which was measured using a continuous treadmill test protocol [14].

### 2.2. Oral Glucose Tolerance Test, Resting Metabolic Rate, Glucose Clamp and Skeletal Muscle Biopsies

For two weeks prior to testing, participants were weight-stabilized (±2% body weight). A registered dietitian created menus and put together coolers complete with two days of meals to distribute to each participant prior to the glucose clamp to manage nutritional intake [15]. All testing was performed in the morning after a 12-h overnight fast, including refraining from caffeine. Participants were given 75 g of glucose to ingest, with samplings repeated at 30-min intervals for 3 h. Plasma glucose was determined by the glucose oxidase method (YSI, 2300 Stat Plus, Yellow Springs, OH, USA) and insulin was measured by radioimmunoassay (Millipore, St. Charles, MO, USA). Area under the curve (AUC) was calculated for both glucose and insulin. Resting metabolic rate (RMR) was measured via indirect calorimetry using COSMED (Quark PFT Ergo, COSMED, Vigevano, Italy) with breath-by-breath gas exchange measurements of oxygen consumption (VO_2_) and carbon dioxide production (VCO_2_), along with other ventilatory parameters. Measurements were performed for 30 min as participants reclined in a supine position beneath a flow-dilution canopy hood in a comfortable, dimly lit, quiet thermo-neutral environment. The Quark RMR metabolic cart was calibrated before each test, and the data were filtered every 30 s. Energy expenditure was calculated by the Weir equation [16] and expressed per 24 h. Data are missing from five participants in each group due to scheduling conflicts. A 3-h (80 mU·m^2^·min^−1^) hyperinsulinemic-euglycemic clamp [17] with two percutaneous needle (5 mm Bergstrom, Stille, Solna, Sweden) vastus lateralis skeletal muscle biopsies under local anesthesia was conducted, one in the basal condition and the second at 120 min of the clamp. Insulin (Humulin, Eli Lilly Co., Indianapolis, IN, USA) (10 min priming and continuous for 180 min) and 20% glucose solution (starting at 10 min for 180 min) were infused. The calculation for M (=glucose utilization) and the procedure for the clamps and blood processing have been previously described [18].

### 2.3. Laboratory Methods

Muscle samples were frozen immediately in clamps cooled in liquid nitrogen and stored at −80 °C until assay. Total RNA of skeletal muscle was isolated using the TRIZOL method. One µg of total RNA was reverse transcripted into cDNA following the Transcriptor First Strand cDNA Synthesis Kit (Cat# 04 896 866 001, Roche Applied Science, Branford, CT, USA) manual. qPCR and data analysis were performed by our routine laboratory method [19]. The CNTF primer/probe was from Thermo Fisher Scientific (Waltham, MA, USA) with the Assay ID# of Hs00173456_m1. Also, 36B4 was used as an internal control for data normalization (36B4 assay ID of Hs99999902_m1).

### 2.4. Interventions

To maintain the subject number in the weight loss classes and exercise group, participants were randomly recruited and assigned to a group intervention of WL or AEX (uncontrolled two-arm intervention study). Participants in the WL group maintained the American Heart Association (AHA) diet and reduced calories by 250–350 kcal/day. They attended weekly group weight loss classes for six months led by a registered dietitian for instruction in the principles of a hypocaloric diet according to therapeutic lifestyle changes (TLC) diet guidelines [20]. Diets were monitored by 7-day food records (or 24-h recalls) using the American Diabetes Association exchange list system. Those participants in the AEX group walked on motorized treadmills where the intensity of exercise began at 50–60% of heart rate reserve (HRR) for 30–40 min during week 1, 55–65% HRR for 45–50 min at week 2, 60–65% HRR for 50 min at week 3, and 65–75% HRR at week 4. By weeks 5–8, they exercised for 50 min at ~70–80% HRR and continued for 6 months. Each exercise session included a 5- to 10-min stretching and warm-up phase and a 5- to 10-min cooldown phase. Heart rates were monitored during exercise training sessions using heart rate monitors (Polar Electro Inc., Lake Success, NY, USA). All testing was performed 36–48 h after the last exercise bout for the AEX group.

### 2.5. Statistical Analyses

Baseline descriptive statistics of the entire group were determined, including mean, standard deviation, and range. Only those who had skeletal muscle CNTF measured were included in the analytical sample for the total group. Sample sizes for certain outcomes were reduced due to scheduling conflicts, technological issues, difficulty in IV-line placement, and participant attrition during WL or AEX due to lack of time, medical reasons unrelated to the study, or declining to continue. The WL group was directly compared to the AEX group using Student’s *t*-test (unpaired) on baseline characteristics, within-group change scores (paired measures *t*-tests), and between-group change scores on measures between baseline and 6 months. Normality was examined using the Shapiro–Wilk test. Non-parametric tests, specifically Spearman’s correlation coefficients, were used to assess relationships between key variables. Statistical significance was set at a two-tailed *p* < 0.05. Data were analyzed using SPSS v.29 (SPSS Inc., Chicago, IL, USA); results are expressed as mean ± SD.

## 3. Results

Participants (*n* = 30 males, *n* = 37 females) were, on average, 61 years of age, classified with obesity by BMI and with low fitness by VO_2_max (Table 1). Skeletal muscle CNTF mRNA expression was measured prior to the interventions (*n* = 67) (Table 1). Baseline skeletal muscle CNTF expression was not associated with age (r = −0.026, *p* = 0.84), BMI (r = −0.010, *p* = 0.94), % body fat (r = 0.142, *p* = 0.27), VO_2_max (r = 0.101, *p* = 0.42), and FFM (r = −0.172, *p* = 0.18). CNTF was inversely correlated with RMR (r = −0.417, *p* = 0.013) and fat oxidation (g/d: r = −0.359, *p* = 0.034).

**Figure 1:**
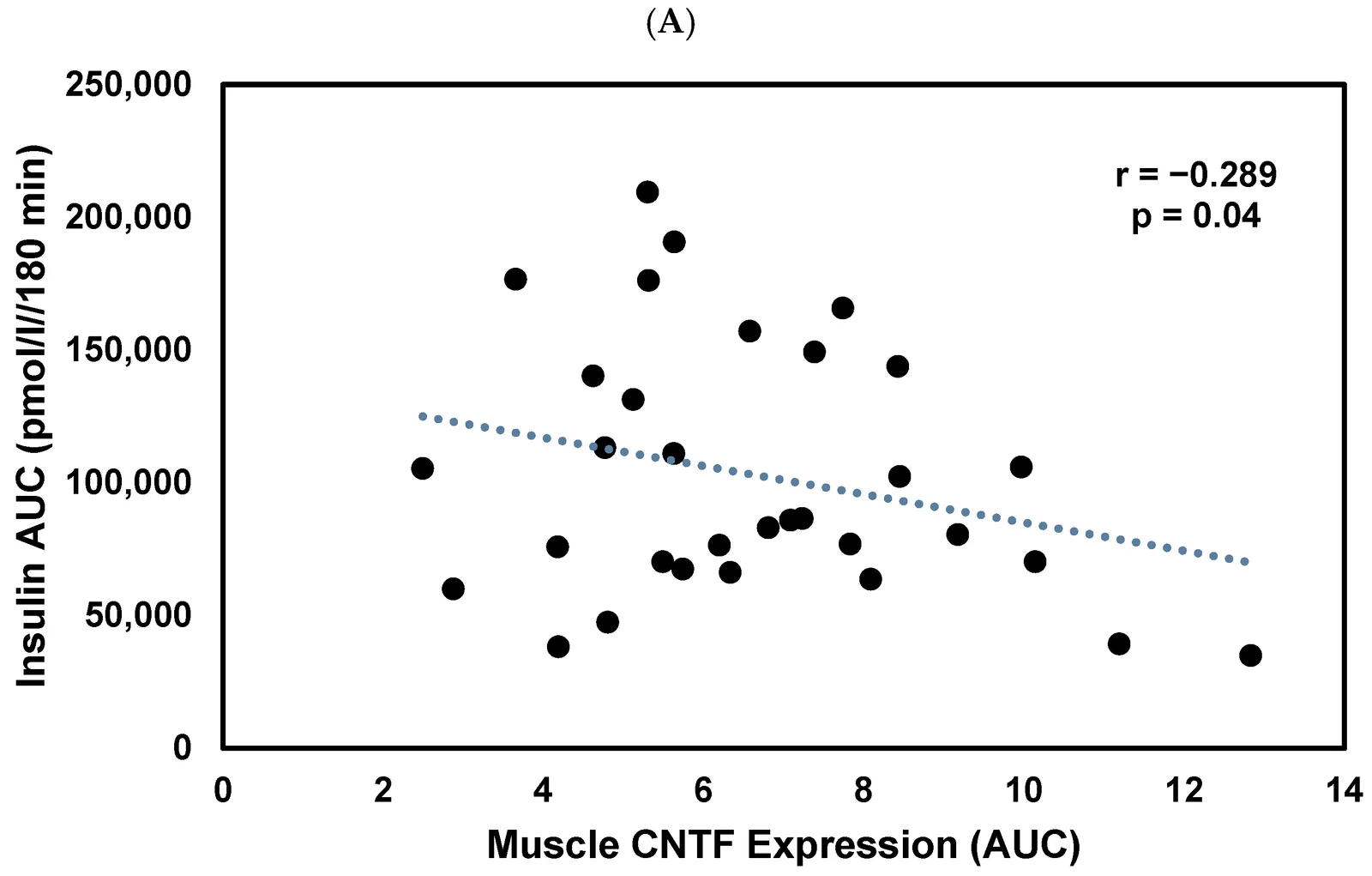

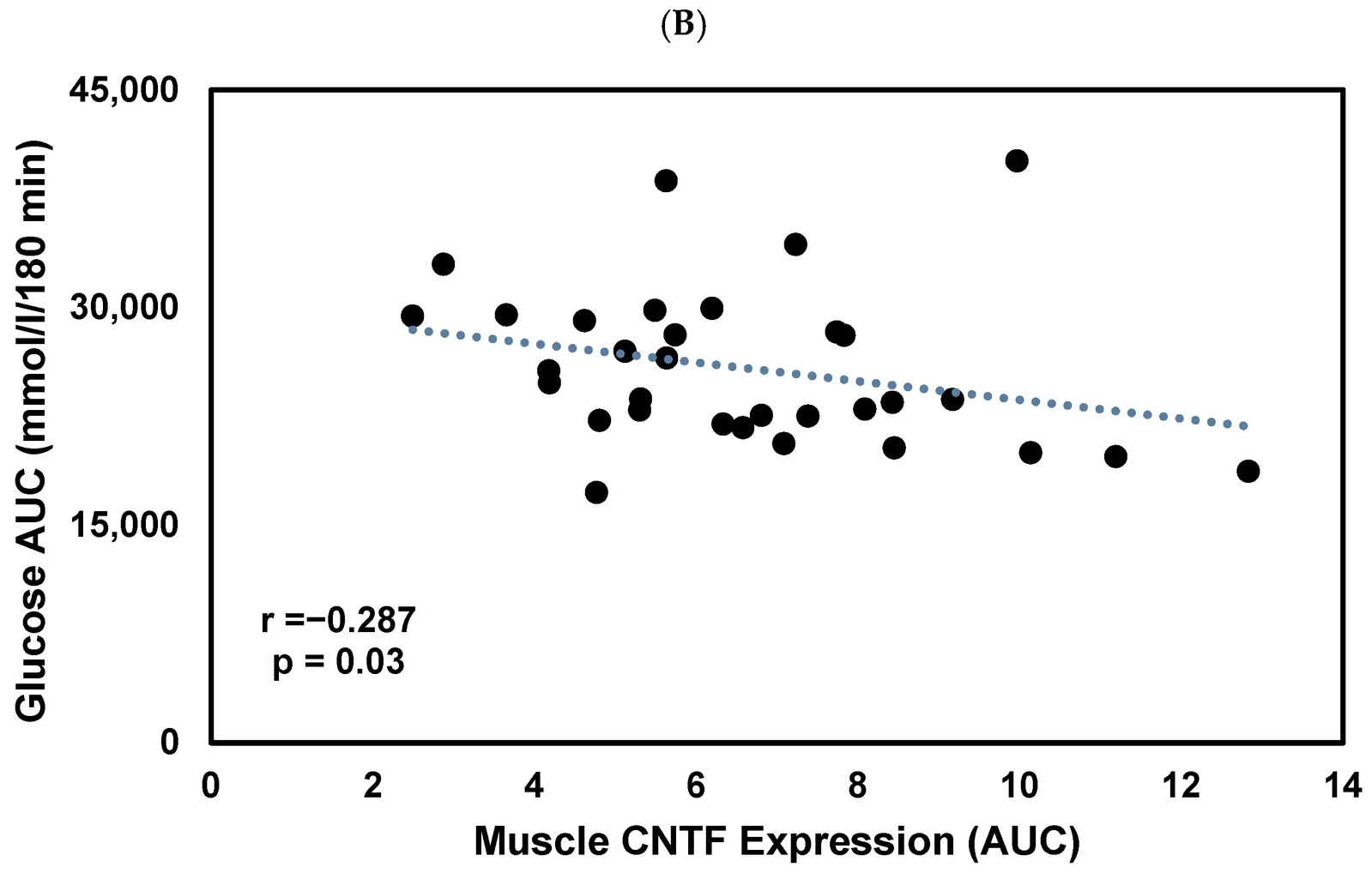
Skeletal muscle CNTF mRNA expression is inversely associated with insulin area under the curve (**A**). Skeletal muscle CNTF mRNA is inversely associated with glucose area under the curve (**B**).

Muscle CNTF expression was inversely associated with 3-h insulin AUC (r = −0.289, *p* = 0.04, Figure 1A) and 3-h glucose AUC (r = −0.287, *p* = 0.03, Figure 1B) but did not reach significance with M or insulin sensitivity (r = 0.229, *p* = 0.08).

Muscle CNTF did not change from basal to hyperinsulinemia (6.46 ± 2.84 vs. 6.67 ± 2.22 AU, *n* = 60, *p* = 0.42, Table 1). Muscle CNTF was not related to inflammatory cytokines at baseline in the total group or by sex (Table 2).

Of this sample, 41 completed WL (*n* = 21) and AEX (*n* = 20) interventions with measurement of skeletal muscle CNTF (Table 3). There were between-group differences in changes in body weight (*p* < 0.001), BMI (*p* < 0.001), and VO_2_max (*p* < 0.001), with a reduction in body weight (*p* < 0.001) and BMI (*p* < 0.001) with WL and increased VO_2_max (*p* < 0.001) with AEX. There was a reduction in %fat, glucose AUC, and insulin AUC (all *p* < 0.01) after WL. %fat decreased (*p* < 0.05), and body weight, glucose AUC, and insulin AUC did not change after AEX.

RMR increased after AEX (*p* < 0.05) and did not change after WL (*p* = 0.28) (Table 4). Percent fat oxidation increased after WL (*p* = 0.006) with a trend toward increased fat utilization (g/min) (*p* = 0.06) after AEX. There were no significant changes in carbohydrate oxidation in either group. M increased after WL (*p* < 0.01) and AEX (*p* < 0.01), and the improvements were not different between groups.

**Figure 2:**
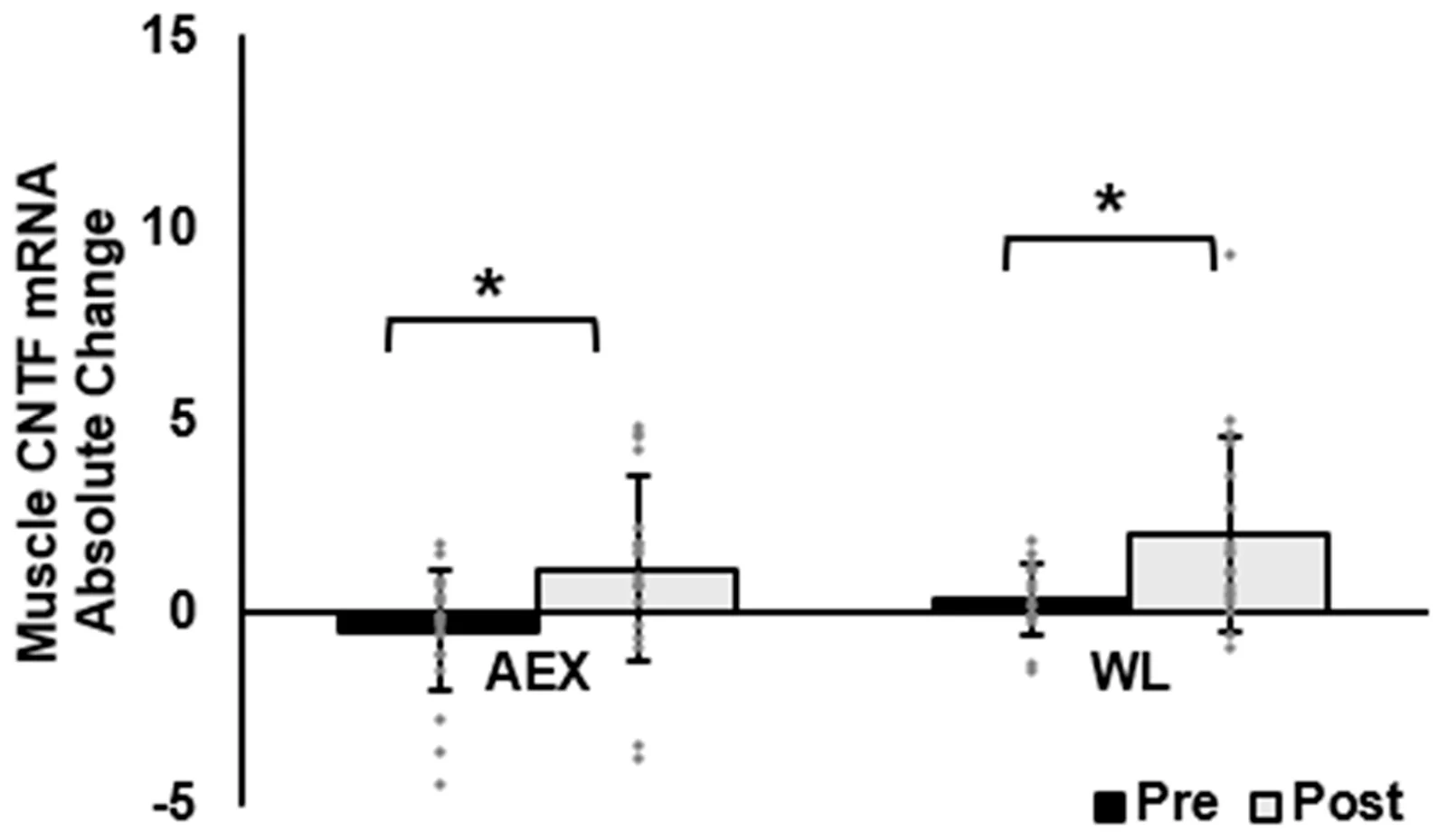
Change in muscle CNTF expression in response to hyperinsulinemia (insulin−basal) pre- and post-weight loss and aerobic exercise training. * *p* < 0.05.

As in the larger sample above, there was no significant difference in basal vs. insulin-stimulated muscle CNTF before AEX (*p* = 0.31) or WL (*p* = 0.14). In comparison of basal vs. insulin-stimulated muscle CNTF after the interventions, there were increases in muscle CNTF in AEX (*n* = 17) (*p* = 0.053) and WL (*n* = 19) (*p* = 0.006) groups resulting in significant change in the response to hyperinsulinemia (e.g., insulin minusbasal) for muscle CNTF (Δ pre vs. Δ post) with AEX (*p* = 0.024) and WL (*p* = 0.011) (Figure 2). In the total group, the change in M was related to the change in muscle CNTF with insulin post (r = 0.36, *p* = 0.046).

## 4. Discussion

Our findings indicate that skeletal muscle CNTF in older adults is associated with glucose intolerance but is not associated with body composition or aerobic fitness in older adults. In vivo insulin stimulation during a glucose clamp did not change muscle CNTF expression in the larger group (*n* = 60) or within the AEX or WL groups prior to the interventions. Yet, after both AEX and WL, there were increases in muscle CNTF expression in response to in vivo hyperinsulinemia. Muscle CNTF mRNA expression decreases after 6 months of weight loss but does not change with the same duration of exercise training. Improvements in insulin sensitivity are associated with increases in muscle CNTF during hyperinsulinemia. Further work is needed to determine the mechanism for muscle CNTF change during hyperinsulinemia after weight loss and exercise training, and its role in glucose metabolism and obesity in older adults.

The oral glucose tolerance test (OGTT) detects glucose intolerance in that the area under the curve is an index of whole glucose excursion after glucose loading and can reflect glycemic index [21]. Our results show that muscle CNTF is negatively associated with both glucose and insulin AUC. These findings align with animal work where CNTF improves glucose uptake and lowers blood glucose after a glucose challenge in lean mice [3,8]. In diet-induced obesity models, CNTF improves hyperinsulinemia and hyperlipidemia [22]. Using the gold standard for the determination of insulin sensitivity (e.g., glucose clamp), muscle CNTF tended to be associated with M prior to the interventions. These results suggest that those with higher skeletal muscle CNTF had better glucose tolerance, glycemic control, beta cell function, and reduced insulin resistance. We are not aware of any reports that have examined both basal and insulin-stimulated CNTF in humans. Intraperitoneal injection of CNTF in mice increases glucose uptake in isolated muscles in a time- and dose-dependent manner [8]. Further, a two-hour intralipid infusion preceded by recombinant CNTF treatment prevented the lipid-mediated reduction in insulin-stimulated glucose uptake in rats [23]. These studies in rodent models led us to test whether CNTF would be altered during a glucose clamp. In our study, CNTF expression did not change during hyperinsulinemia prior to the interventions, but after the interventions, it did increase along with increased muscle glucose uptake during hyperinsulinemia. These results, as well as our finding of the association of the change in muscle CNTF during hyperinsulinemia with improvement in insulin sensitivity (M), suggest that muscle CNTF may play a role in the changes in glucose metabolism with weight loss and exercise.

We also examined whether CNTF was related to resting metabolic rate given that CNTF treatment increases basal metabolic rate in db/db mice [24]. CNTF was inversely related to both RMR and fat oxidation, which contrasts with work in animal models where CNTF enhances fatty acid oxidation in muscle of mice [25]. We observed an increase in RMR after AEX and no change after weight loss. Interestingly, studies that include direct measurement of RMR by indirect calorimetry and combine aerobic and resistance training have reported a decline in RMR in older women [26] or lack of significant change [27]. Our results did not show relationships between circulating inflammatory markers and muscle CNTF levels. Infusion of IL-6 increases glucose uptake and fatty acid oxidation in human skeletal muscle [28] and in mice through the PI3-kinase/Akt pathway [8]. In addition, IL-6 increases AMPK, and it may play a role in energy utilization during exercise and recovery and substrate metabolism [29,30]. The signaling of Il-6 and other muscle-derived cytokines or myokines is complex, and a recent review synthesized the evidence of myokines with exercise, aging, and disease states [31].

Determination of CNTF levels directly in skeletal muscle is more challenging due to the invasive nature of a muscle biopsy than measuring circulating levels, and only a few studies have measured muscle CNTF in response to exercise training. A recent study demonstrated that serum CNTF was greater in those with higher BMI and among females compared to males, whereas muscle CNTF expression did not differ based on sex [32]. After six months of aerobic, resistance, or combined exercise compared to control, plasma CNTF significantly decreased, but in skeletal muscle, there was a sex-specific response to the interventions such that there was a reduction in muscle CNTF expression in males compared to females. Our study was unable to examine sex differences due to the number of individuals by sex per intervention group. However, our prior work shows baseline sex differences in skeletal muscle metabolism, specifically the change in glycogen synthase fractional and independent activity and AKT protein expression with insulin stimulation [33]. Moreover, basal AMPK, GSK-3β, ERK1/2, and p38 are lower in older men than in older women, and changes in glycogen synthase fractional activity with insulin stimulation are higher in women than in men after exercise training and higher in men than women after weight loss [33]. Thus, it is possible that there are sex differences in muscle CNTF and response to exercise or weight loss, but that is yet to be determined.

In a randomized controlled trial of women with multiple sclerosis, there was no change in circulating CNTF after a 12-week multimodal exercise program (resistance, endurance, Pilates, balance and stretching exercises) [34]. Although their study did not measure skeletal muscle CNTF, their finding of a lack of change in circulating CNTF agrees with our report that basal mRNA CNTF in skeletal muscle did not significantly change with exercise training. Yet, differences in study population, disease status, exercise modality, duration of the intervention, and tissue versus circulating measurements make comparisons between our study and this one difficult.

Limitations of the study include the absence of measurement of circulating CNTF levels or protein levels to add insight into the role of CNTF in glucose metabolism and a more comprehensive biological interpretation of our findings. Our study included a sample of adults with overweight and obesity, limiting its applicability to normal weight individuals. Additionally, there is a risk of Type 1 error with the small sample size and multiple testing. Strengths of the study include the measurement of CNTF in skeletal muscle requiring a muscle biopsy, its detection during in vivo insulin stimulation, and extensive phenotyping of study participants. Furthermore, the long duration of the interventions and controlled conditions are study strengths. Future studies with a larger sample size could address whether sex modifies the response to weight loss and aerobic training.

## 5. Conclusions

CNTF mRNA expression in skeletal muscle is related to glucose tolerance but does not appear to be associated with body composition or fitness. The relationship between CNTF gene expression and glucose tolerance may be helpful for aged adults for muscle health in that CNTF could help muscles respond more effectively to insulin, suggesting that it could affect blood glucose spikes after meals. In addition, our observation that improvements in insulin sensitivity are associated with increases in muscle CNTF during hyperinsulinemia may suggest a role of CNTF in the prevention of or delay in diabetes onset and may also support muscle maintenance and function in older adults, but additional investigations are warranted. Although muscle CNTF mRNA expression is reduced by weight loss, further work is needed to determine its influence on glucose metabolism and obesity in older adults.

## Figures and Tables

**Table 1:** Baseline physical and metabolic characteristics.

	Mean ± SD
Sex (*n*), Male/Female	67 (30/37)
Age (years)	60.75 ± 7.58
Weight (kg)	95.07 ± 17.78
BMI (kg/m^2^)	33.08 ± 5.28
Body Fat (%)	41.98 ± 8.04
Fat Mass (kg)	40.28 ± 12.05
Lean Body Mass (kg)	52.27 ± 11.11
Fat-Free Mass (kg)	55.13 ± 11.55
Absolute VO_2_ (L/min)	2.27 ± 0.66
Relative VO_2_ (mL/kg/min)	23.93 ± 5.50
Glucose AUC (mmol/L/180 min)	26,027.4 ± 6917.7
Insulin AUC (pmol/L/180 min)	101,863.8 ± 51,093.0
M (umol/kg/min)	26.2 ± 10.9
M (umol/kgFFM/min)	44.6 ± 19.4
Muscle CNTF Basal (AU)	6.34 ± 2.79
Muscle CNTF Insulin Stimulated (AU)	6.67 ± 2.22

BMI = body mass index, AUC = area under the curve, CNTF = ciliary neurotrophic factor. Values are mean ± SD. N = 61 for glucose clamps (M).

**Table 2:** Relationships between basal muscle CNTF expression and plasma inflammatory cytokines.

	All (N = 67)	Males (N = 30)	Females (N = 37)
IL1B	−0.121	−0.301	−0.120
IL6	0.103	−0.100	0.140
IL8	−0.008	−0.185	−0.011
TNFα	−0.057	−0.130	−0.011

IL = interleukin, TNF = tumor necrosis factor.

**Table 3:** Physical and metabolic characteristics before and after weight loss (WL) and aerobic exercise training (AEX).

	WL			AEX		
Age (Years)	60.2 ± 6.5			61.7 ± 8.2		
*n* (Male/Female)	21 (11/10)			20 (8/12)		
	Pre	Post	[95% confidence intervals]	Pre	Post	[95% confidence intervals]
BMI (kg/m^2^)	35.1 ± 4.4	31.9 ± 4.3 *	[−2.8, −3.6]	31.9 ± 5.4	31.5 ± 6.0	[0.9, 1.0]
Weight (kg)	101.7 ± 18.3	92.5 ± 16.5 *	[−7.8, −10.8]	93.7 ± 17.4	94.4 ± 18.8	[2.6, −2.8]
Body Fat (%)	43.7 ± 7.2	39.4 ± 8.4 *	[−1.6, −4.3]	36.5 ± 7.4	35.2 ± 8.6 *	[−0.4, −2.6]
Fat Mass (kg)	44.3 ± 10.1	37.0 ± 10.5	[−5.1, −8.5]	34.9 ± 11.0	33.9 ± 13.0	[0.3, −3.0]
Lean Body Mass (kg)	54.5 ± 12.5	53.9 ± 12.1	[−0.7, −2.3]	56.6 ± 9.9	57.5 ± 11.0	[1.8, −0.2]
Fat-Free Mass (kg)	57.4 ± 13.0	56.9 ± 12.6	[−0.7, −2.3]	59.5 ± 10.4	60.6 ± 11.2	[1.8, 0.0]
VO_2_ (L/min)	2.40 ± 0.57	2.34 ± 0.60	[0.0, −0.1]	2.46 ± 0.61	2.79 ± 0.63 *	[0.4, 0.2]
VO_2_ (mL/kg/min)	23.6 ± 5.0	25.5 ± 5.8	[2.3, 0.8]	26.1 ± 4.8	30.1 ± 6.3 *	[5.7, 2.7]
Glucose AUC (mmol/L/180 min)	26,094.2 ± 5416.8	22,092.5 ± 6673.6 *	[−2411.3, −5974.4]	25,490.4 ± 5767.0	25,163.6 ± 5831.6	[1678.2, −2318.8]
Insulin AUC (pmol/L/180 min)	110,283.3 ± 49,459.2	76,830.4 ± 31,999.6 *	[−8839.9, −49,972.6]	93,908.2 ± 45,458.5	88,042.1 ± 40,347.8	[88.1, −17,598.1]
M (umol/kg/min)	21.9 ± 8.8	31.7 ± 10.2 †	[13.7, 5.0]	27.2 ± 11.4	33.0 ± 12.8 †	[9.6, 2.4]
M (umol/kgFFM/min)	39.7 ± 19.1	52.9 ± 19.2 †	[19.2, 5.3]	44.9 ± 19.9	52.8 ± 5.6 *	[14.4, 2.2]
Muscle CNTF Basal (AU)	6.10 ± 1.89	4.90 ± 1.42	[−0.36, −2.3]	6.70 ± 2.45	6.37 ± 1.88	[0.8, −1.4]
Muscle CNTF Insulin Stimulated (AU)	6.45 ± 2.22	6.73 ± 2.85	[1.4, 0.8]	6.31 ± 1.80	7.52 ± 2.57	[2.2, −0.1]

BMI = body mass index, AUC = area under the curve, CNTF = ciliary neurotrophic factor, AU = arbitrary units. Values are mean ± SD. * *p* < 0.05, † *p* < 0.01.

**Table 4:** Resting energy expenditure and substrate oxidation.

	WL (*n* = 16)		[95% Confidence Intervals]	*p*-Value	AEX (*n* = 15)		[95% Confidence Intervals]	*p*-Value
	Pre	Post			Pre	Post		
RMR (kcal/day)	1489 ± 323	1568 ± 375	[227, −70]	0.277	1537 ± 343	1660 ± 297 *	[236, 11]	0.034
CHO (g/min)	0.02 ± 0.04	0.00 ± 0.04	[0.01, −0.05]	0.238	0.04 ± 0.05	0.01 ± 0.06	[0.01, −0.06]	0.167
CHO Ox (%)	11.25 ± 10.00	9.56 ± 8.32	[5.61, −8.98]	0.629	13.71 ± 14.82	12.32 ± 13.45	[9.13, −11.92]	0.781
Fat (g/min)	0.08 ± 0.03	0.09 ± 0.03	[0.02, −0.01]	0.490	0.08 ± 0.02	0.10 ± 0.03	[0.03, 0.00]	0.062
Fat Ox (%)	74.41 ± 13.11	85.94 ± 10.41	[19.21, 3.85]	0.006	71.93 ± 12.81	76.87 ± 13.14 ^†^	[11.57, −1.70]	0.133

RMR = resting metabolic rate, CHO = carbohydrate, Ox = oxidation. Data are missing from five participants in each group due to scheduling conflicts, technological issues, or difficulty following procedures (e.g., not fasting). Values are mean ± SD. * *p* < 0.05, † *p* < 0.01.

## Data Availability

Data will be available upon reasonable request.

## Abbreviations

The following abbreviations are used in this manuscript:

AEX	Aerobic exercise training
AHA	American Heart Association
AUC	Area under the curve
BMI	Body mass index
CRP	C-reactive protein
CNTF	Ciliary neurotrophic factor
DXA	Dual-energy X-ray absorptiometry
FFM	Fat-free mass
HRR	Heart rate reserve
HOMA	Homeostatic Model Assessment
IL-1β	Interleukin-1 beta
IL-6	Interleukin-6
IL-8	Interleukin-8
JAK	Janus kinase
LIFR	Leukemia inhibitory factor receptor
MAPK	Mitogen-activated protein kinase
OGTT	Oral glucose tolerance test
PI3K	Phosphatidylinositol 3-kinase
qPCR	Quantitative polymerase chain reaction
RMR	Resting metabolic rate
STAT	Signal transducer and activator of transcription
TNF	Tumor necrosis factor
VO_2_	Oxygen consumption
VO_2_max	Maximal oxygen consumption
WL	Weight loss
